# Percutaneous Coronary Interventions: Bleeding Risk Assessment and Management

**DOI:** 10.3390/jcm15145729

**Published:** 2026-07-22

**Authors:** Adil Salihu, David Meier, Thabo Mahendiran, Aurelia Zimmerli, Jeremie Buri, Marine Klopfenstein, Emmanuelle Scala, Stephane Fournier

**Affiliations:** 1 Department of Cardiology, Lausanne University Hospital, University of Lausanne, Rue du Bugnon 46, 1011 Lausanne, Switzerland; david.meier@chuv.ch (D.M.); thabodhan.mahendiran@chuv.ch (T.M.); aurelia.zimmerli@chuv.ch (A.Z.); jeremie.buri@chuv.ch (J.B.); marine.klopfenstein@chuv.ch (M.K.); 2 Department of Anesthesiology, Lausanne University Hospital, University of Lausanne, Rue du Bugnon 46, 1011 Lausanne, Switzerland; emmanuelle.scala@chuv.ch

**Keywords:** blood management, blood transfusion, anticoagulation management, percutaneous coronary intervention

## Abstract

Bleeding is one of the most common and feared complications after coronary angiography and percutaneous coronary intervention (PCI). It is associated with longer hospital stays, higher mortality, and worse clinical outcomes. As the number of patients at high bleeding risk (HBR) continues to grow, preventing bleeding has become an important part of PCI management. This practical review summarizes current evidence on how to assess bleeding risk and reduce bleeding before, during, and after PCI. Several tools, including the BARC classification, ARC-HBR criteria, PRECISE-DAPT, and DAPT scores, help identify patients who may benefit from tailored treatment. Current strategies include the use of radial access, optimized anticoagulation, appropriate selection of antiplatelet therapy, newer-generation drug-eluting stents, and shorter durations of dual antiplatelet therapy in selected HBR patients. We also discuss the management of patients with atrial fibrillation requiring oral anticoagulation, as well as those with anemia or thrombocytopenia. Although significant progress has been made, several questions remain unanswered, particularly regarding transfusion thresholds, antithrombotic therapy in complex patients, and the best balance between bleeding and ischemic risks. Ongoing clinical trials are expected to provide further evidence and help improve the management of patients undergoing PCI.

## 1. Introduction

Bleeding complications remain one of the major challenges in patients undergoing coronary angiography and percutaneous coronary intervention (PCI). Beyond their immediate clinical consequences, bleeding events are strongly associated with prolonged hospitalization, increased healthcare costs, recurrent ischemic events, and mortality [1,2,3,4]. The growing complexity of contemporary PCI, combined with the widespread use of potent antithrombotic therapies and the aging population with multiple comorbidities, has further increased the proportion of patients considered at high bleeding risk (HBR). Current data suggest that up to 40% of patients undergoing PCI may fulfill this definition [2]. This review summarizes current evidence regarding bleeding risk evaluation and prevention strategies before, during, and after PCI, with a particular focus on HBR patients and contemporary antithrombotic management. It also discusses current uncertainties surrounding transfusion strategies and future perspectives in the field of personalized bleeding prevention.

## 2. Bleeding Risk Evaluation

Several tools have been developed to assess bleeding risk in patients undergoing coronary angiography and PCI.

A prerequisite to the interpretation of these risk stratification tools is a clear understanding of the Bleeding Academic Research Consortium (BARC) bleeding classification, which categorizes bleeding events into six grades, as shown in Table 1, ranging from type 0 to type 5. BARC type 0 indicates the absence of any bleeding episode. Type 1 describes minor bleeding that does not prompt medical attention, hospitalization, or specific treatment and is generally considered clinically insignificant. Type 2 includes overt bleeding events that require medical evaluation, diagnostic testing, therapeutic intervention, or hospitalization, but do not fulfill the criteria for severe bleeding. Type 3 represents major bleeding and includes events associated with a substantial decline in hemoglobin levels, the need for blood transfusion, cardiac tamponade, surgical management of bleeding, hemodynamic compromise requiring intravenous vasoactive agents, or critical-site hemorrhages such as intracranial or vision-threatening ocular bleeding. Type 4 is specifically related to coronary artery bypass grafting (CABG) and encompasses significant perioperative bleeding complications, including reoperation for hemostasis, large-volume transfusions, intracranial hemorrhage, or excessive postoperative chest tube drainage. Type 5 refers to fatal bleeding events in which hemorrhage is considered the direct cause of death and may be classified as probable or definite depending on the available clinical, imaging, or pathological evidence [5]. Among these, BARC type 3 and type 5 bleeding events are the most frequently used endpoints in clinical studies, with type 3 being an independent predictor of mortality [6]. This standardized classification has also enabled the identification and definition of patients at high bleeding risk (HBR) based on 20 major and minor clinical criteria. The Academic Research Consortium (ARC) has defined HBR as an estimated risk of BARC type 3 or 5 bleeding ≥ 4% at 1 year or a risk of intracranial hemorrhage ≥ 1% at 1 year [7]. The BARC classification remains the preferred bleeding definition owing to its standardized framework; however, other classifications, such as TIMI and GUSTO, have demonstrated comparable prognostic value for mortality prediction [6].

When it comes to bleeding risk assessment, the PRECISE-DAPT score (PREdicting bleeding Complications In patients undergoing Stent Implantation and SubsequEnt Dual AntiPlatelet Therapy), originally developed to estimate the risk of out-of-hospital bleeding in patients receiving dual antiplatelet therapy (DAPT) is widely used. This score incorporates five variables: age, creatinine clearance, hemoglobin level, white blood cell count, and prior spontaneous bleeding. A score ≥ 25 identifies patients at high bleeding risk who may benefit from a shortened duration of DAPT, whereas a score < 25 suggests that standard or prolonged DAPT (>12 months) may be considered without a substantial increase in bleeding risk [8]. The PRECISE-DAPT score should therefore be calculated before every procedure to allow a better assessment of bleeding risk in our patients.

In contrast to the two previous scores, which mainly focus on bleeding risk, the DAPT score helps guide the decision regarding extended DAPT (DAPT score ≥ 2), provided that no major bleeding event has occurred during the initial DAPT period, as demonstrated in the DAPT trial [9]. More recently, a randomized study prospectively evaluated DAPT duration adjustment according to the DAPT score compared with a standard strategy in both acute coronary syndrome (ACS) and chronic coronary syndrome (CCS) patients. This tailored approach was associated with a reduction in net adverse clinical events, including all-cause death, myocardial infarction, urgent target vessel revascularization, and major bleeding, mainly driven by a reduction in ischemic events, without an increase in bleeding risk compared with standard therapy [10].

Identification of a patient as being at HBR allows anticipation and adaptation of several aspects of management, including peri-procedural strategies, stent selection, and the duration of DAPT. Although these scores may be useful, guideline recommendations supporting their use remain low.

## 3. Bleeding Prevention

### 3.1. Vascular Access

Guidelines for ACS recommend preferential use of radial access over femoral access [11,12]. A meta-analysis based on seven randomized clinical trials demonstrated a reduction in all-cause mortality and major bleeding with radial access compared with femoral access in ACS. This benefit is largely attributable to a lower risk of access-site complications [13]. The use of distal radial or ulnar access is associated with outcomes comparable to conventional radial access, according to two meta-analyses, making it a reasonable alternative. However, no comparisons have been performed with femoral access [14,15].

In cases where radial access is not feasible, several strategies have been shown to mitigate vascular complications associated with femoral access. Among these strategies, ultrasound-guided femoral puncture has been associated with a significant reduction in major vascular complications or major bleeding, although this benefit appears to be limited to patients in whom a vascular closure device was used, as demonstrated in a meta-analysis [16]. Overall, this approach improves first-pass success rates and reduces the number of puncture attempts [17].

While vascular closure devices consistently demonstrate faster time to hemostasis and earlier ambulation compared with manual compression, evidence regarding their impact on bleeding and vascular complications shows no significant difference overall, according to a systematic review [18]. However, subgroup analyses suggest a reduction in bleeding risk among high-risk populations, including women, patients with chronic kidney disease, and those receiving high-potency P2Y12 inhibitors (ticagrelor or prasugrel) [18].

### 3.2. Management of Anticoagulant and Antiplatelet Therapy

#### 3.2.1. Pre-Procedural

Recommendations on anticoagulation management before PCI have been previously established. In the setting of atrial fibrillation, no bridging therapy is recommended in patients receiving chronic oral anticoagulation [19,20]. Available data even suggest an increased bleeding risk associated with bridging strategies [21].

**Figure 1:**
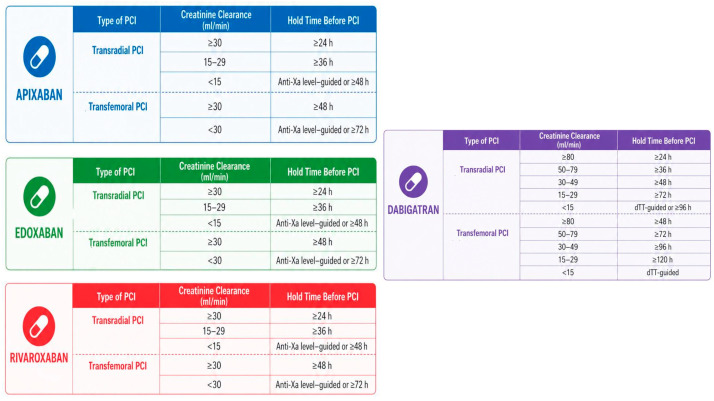
Timing of DOAC Interruption Before PCI. DOAC: direct oral anticoagulant; dTT: diluted thrombin time; PCI: Percutaneous coronary intervention.

In emergency situations, the procedure should be performed regardless of ongoing anticoagulation therapy, and routine reversal of anticoagulation is not recommended. Adjustments to anticoagulation are only considered in urgent or elective procedures. For patients treated with vitamin K antagonists (VKA), the intervention is recommended once the international normalized ratio (INR) is <2.0. In patients receiving direct oral anticoagulants (DOACs), temporary interruption is recommended, with the duration depending on renal function, the specific molecule used, and the vascular access site, as illustrated in Figure 1 adapted from the American Heart Association (AHA) recommendations [22].

However, a meta-analysis comparing interruption with or without bridging versus continuation of VKA therapy before coronary angiography with or without PCI demonstrated no significant difference in major bleeding outcomes [23]. This approach is further supported by recommendations from the Society for Cardiovascular Angiography & Interventions (SCAI) when coronary angiography is planned via radial access [24]. Similarly, the DOAC-NOSTOP trial was a randomized study designed to assess the 30-day risk of major bleeding in patients undergoing coronary angiography with or without PCI via radial access while continuing DOAC therapy uninterrupted. The study confirmed the safety of performing these procedures under ongoing anticoagulant treatment [25]. Such recommendations do not apply when a femoral access procedure is planned or after a failed radial access and are not recommended by SCAI. Although the literature reports a higher risk of hemorrhagic and vascular complications with the femoral approach in anticoagulated patients, no specific data are available regarding the potential benefit of ultrasound guidance in this setting. However, if the procedure cannot be deferred, ultrasound-guided femoral access may help mitigate these risks [16].

While SCAI guidelines recommend a bridging strategy without further nuance for patients with mechanical heart valves, the European guidelines on the management of patients undergoing non-cardiac surgery provide additional guidance. According to these recommendations, bridging anticoagulation should be considered in patients with a mechanical aortic valve replacement associated with at least one thromboembolic risk factor (including atrial fibrillation, prior thromboembolism, severe left ventricular dysfunction, or a hypercoagulable state), as well as in those with an older-generation mechanical aortic valve replacement or a mechanical mitral valve replacement, given their inherently higher thromboembolic risk profile [24,26].

Regarding pretreatment with a second antiplatelet agent in addition to aspirin, current guidelines recommend, albeit with a low level of supporting evidence, administering a loading dose only in patients with non-ST-elevation myocardial infarction (NSTEMI) when the delay is expected to exceed 24 h [11]. Indeed, a meta-analysis of seven randomized controlled trials demonstrated that routine DAPT pretreatment, compared with loading after coronary anatomy was defined, increased 30-day major bleeding without improving cardiovascular outcomes [27]. In contrast, no reduction in bleeding risk has been demonstrated with pretreatment compared with no pretreatment in patients presenting with ST-elevation myocardial infarction (STEMI) [28]. Regarding the choice of P2Y12 inhibitor, clopidogrel has the most favorable bleeding profile compared with prasugrel and ticagrelor. In a meta-analysis including more than 15 randomized controlled trials assessing efficacy and safety of P2Y12 inhibitors after PCI, ticagrelor was associated with a significantly higher risk of major bleeding than clopidogrel [29]. Ticagrelor and prasugrel remain the preferred treatment options for patients with ACS; however, clopidogrel is an appropriate alternative in patients at HBR. Currently, no adjustment of the loading dose of P2Y12 inhibitors is recommended for patients at HBR [11,12,30].

#### 3.2.2. During Procedure

Anticoagulation during PCI remains mandatory because of the risk of thrombosis, but it can also increase the risk of bleeding, including both access-site and non-access-site bleeding. Two drugs are primarily used: unfractionated heparin (UFH) and bivalirudin. UFH remains the standard of care according to European Society of Cardiology (ESC) and AHA guidelines, regardless of the vascular access site. Bivalirudin is a direct thrombin inhibitor with the advantage of having a shorter half-life and can be used in patients with a history of heparin-induced thrombocytopenia. Bivalirudin is primarily eliminated through cleavage by thrombin, its main mechanism of clearance. This elimination is enhanced in areas of blood stagnation, where increased thrombin activity accelerates drug degradation, a phenomenon notably demonstrated in cardiac surgery settings [31]. Although a higher rate of suboptimal flow (TIMI ≤ 2) has been reported with bivalirudin compared to heparin, this does not appear to translate into an increased risk of stent thrombosis [32].

The BRIGHT-4 trial compared bivalirudin (with a full-dose infusion for 2–4 h post-procedure) to UFH in patients with STEMI. It showed a reduction in the composite endpoint of all-cause mortality and BARC 3–5 bleeding compared with heparin alone. This benefit was mainly driven by a reduction in non-access-site bleeding, as most procedures were performed via radial access [33]. However, this remains the only study to demonstrate such a benefit for radial access [34]. Another randomized trial, the VALIDATE-SWEDEHEART trial, which included 6006 patients with STEMI or NSTEMI, predominantly treated via radial access, found no difference in the composite endpoint of all-cause mortality, myocardial infarction, or major bleeding at 180 days when comparing bivalirudin and heparin [35].

Nevertheless, a difference emerges when comparing these two agents in the setting of femoral access. Studies have shown a higher bleeding risk with heparin compared with bivalirudin, initially driven by the more frequent use of glycoprotein IIb/IIIa inhibitors, which has declined over time [36]. More recent individual patient-level meta-analyses of randomized trials comparing heparin and bivalirudin in patients with NSTEMI, of whom more than 60% underwent procedures via femoral access, have shown no significant difference in ischemic outcomes. However, bivalirudin was associated with a lower risk of major bleeding, including both access-site and non-access-site bleeding [37]. This is reflected in both ESC and AHA guidelines, which suggest the use of bivalirudin as an alternative anticoagulant in both STEMI and NSTEMI to reduce bleeding risk [11,12].

In cases of periprocedural bleeding where heparin has been administered, the use of protamine may be considered on a case-by-case basis. The main concern associated with protamine reversal is the potential risk of stent thrombosis. However, a meta-analysis including 11 studies comparing protamine use with standard care did not demonstrate any increase in the risk of stent thrombosis or mortality, while showing a reduction in major bleeding [38]. Nevertheless, these findings are only applicable to patients receiving DAPT pretreatment. There are no robust data supporting the use of protamine in patients without antiplatelet pretreatment, and its use should therefore be reserved for cases of life-threatening hemorrhage.

Routine use of glycoprotein IIb/IIIa inhibitors is not recommended in current guidelines because of the lack of clinical benefit and the increased risk of bleeding. In cases of no-reflow or a high thrombus burden, the potential ischemic benefit should be carefully weighed against the risk of bleeding before their use [11,12].

#### 3.2.3. Post Procedural

To reduce bleeding risk, both the choice and duration of DAPT are key considerations, and these are also closely linked to the type of stent implanted [39].

Current ESC and AHA guidelines recommend a default DAPT duration of 12 months for ACS. Adjustment of DAPT duration is essential in patients at HBR, but should not come at the expense of increased ischemic risk. A meta-analysis published in European Heart Journal including 11 randomized controlled trials evaluating DAPT duration after PCI demonstrated the ischemic safety of 1- or 3-month DAPT strategies, while showing a reduction in bleeding risk compared with standard-duration DAPT [40]. Ultimately, current guidelines support the use of an ultra-short DAPT strategy in this category of patients [11,12]. A duration of DAPT shorter than one month, followed by P2Y12 inhibitor monotherapy in patients with ACS, is associated with a higher ischemic risk compared with a standard DAPT strategy [41].

Additional strategies have emerged to mitigate bleeding risk, including de-escalation and shortening of antiplatelet therapy. One approach, tailored to the patient’s ischemic risk, involves reducing the duration of DAPT to 3–6 months, followed by continuation of a P2Y12 inhibitor as monotherapy. A meta-analysis of six randomized controlled trials including 24,096 patients with ACS compared standard DAPT with a strategy of short-duration DAPT (1–3 months) followed by P2Y12 inhibitor monotherapy. This analysis demonstrated a significant reduction in major bleeding, without a significant increase in ischemic events [42]. The AHA guidelines go even further, recommending, with a Class I, Level of Evidence A, that aspirin can be discontinued and ticagrelor continued as monotherapy after an initial period of well-tolerated DAPT with aspirin and ticagrelor [12,43,44]. Some authors have taken this concept further by comparing DAPT versus ticagrelor monotherapy initiated immediately after PCI for STEMI. Although the study was underpowered (199 patients) and subject to several limitations, no difference in ischemic outcomes was observed, while a significant reduction in bleeding risk was reported. Further research is needed to confirm the validity of this strategy [45].

In ACS, guideline-recommended DAPT consists of aspirin combined with a potent P2Y12 inhibitor, such as ticagrelor or prasugrel, both of which have demonstrated superior ischemic protection compared with clopidogrel [46,47]. An alternative strategy involves de-escalation of P2Y12 inhibition, by switching to clopidogrel. These approaches have been associated with a lower bleeding risk (BARC ≥ 2) compared with standard regimens using more potent P2Y12 inhibitors, while maintaining non-inferior ischemic outcomes [48,49]. The level of recommendation for this bleeding-reduction strategy remains low in current guidelines, with a Class IIb recommendation.

Similarly, current guidelines for CCS recommend a standard 6 months of DAPT consisting of aspirin and clopidogrel [50,51]. AHA guidelines also support, with moderate recommendation, shortened DAPT strategies (1–3 months) followed by P2Y12 inhibitor monotherapy with prasugrel or ticagrelor for a total duration of 12 months, before resuming aspirin therapy in patients at HBR [51].

Emerging evidence is challenging the conventional long-term use of aspirin in favor of clopidogrel monotherapy. The HOST-EXAM trial compared aspirin versus clopidogrel following a standard course of DAPT, with a mean follow-up of 5.8 years. Clopidogrel demonstrated superiority on both the secondary thrombotic endpoint (7.9% vs. 11.9%; HR 0.66 [95% CI, 0.55–0.79]; *p* < 0.001) and the secondary bleeding endpoint (4.5% vs. 6.1%; HR 0.74 [95% CI, 0.57–0.94]; *p* = 0.016) [52]. These findings further reinforce the positive signal previously observed in the SMART-CHOICE 3 trial [53]. Of note, the HOST-EXAM trial incorporated CYP2C19 loss-of-function testing, and a subanalysis failed to demonstrate a significant difference in the primary endpoint of major adverse cardiac and cardiovascular events based on genetic status. More broadly, these studies did not systematically assess clopidogrel resistance through CYP2C19 genetic testing or platelet function testing after standard DAPT duration. Current AHA and ESC guidelines on the management of ACS and CCS do not advocate for routine testing following a standard course of DAPT. This is consistent with studies that have found no significant difference in ischemic outcomes between unguided and guided de-escalation strategies, whether based on platelet function testing or genetic testing [54].

Several stent classifications exist, although the most recent network meta-analysis categorized stents according to polymer type. Currently, three main categories of stents are routinely used in clinical practice: durable-polymer drug-eluting stents (DP-DES), including the zotarolimus-eluting Resolute Onyx (Medtronic) and everolimus-eluting Xience (Abbot Vascular) stents; biodegradable-polymer drug-eluting stents (BP-DES), including the sirolimus-eluting Orsiro (Biotronik) and everolimus-eluting Synergy (Boston Scientific) stents; and polymer-free drug-coated stents (PF-DCS), represented by the biolimus A9-coated BioFreedom (Biosensors) stent.

This recent network meta-analysis including 13 randomized clinical trials enrolling patients at HBR compared different stent platforms together with standard versus short DAPT duration (<3 months) in this specific population. The analysis demonstrated that DP-DES provided the most favorable balance between ischemic and bleeding risk. However, all short-duration DAPT strategies were associated with a more favorable bleeding profile compared with standard DAPT [55]. These findings are consistent with a previous Bayesian meta-analysis of randomized controlled trials including 43,875 patients, in which DP-DES were associated with significantly lower rates of major bleeding compared with BP-DES in the setting of short-term DAPT [56].

Drug-coated balloons (DCBs) represent also a safe and valuable treatment option, particularly in patients at HBR [57]. Similar to DES, shortened DAPT strategies have been validated following DCB angioplasty. A meta-analysis comparing 1 month versus 3–6 months of DAPT demonstrated that abbreviated DAPT is both safe and effective [58]. Accordingly, the current International DCB Consensus Group recommends 4 weeks of DAPT in patients with HBR [59]. In selected patients with an exceptionally high bleeding risk, DAPT may be further shortened to 2 weeks or, in extreme cases, a single antiplatelet therapy strategy may be considered [57].

A substantial proportion of patients undergoing PCI have atrial fibrillation and require combined antithrombotic therapy, including DAPT and oral anticoagulation with either a DOAC or VKA. Whenever possible, DOAC should be preferred over VKA because it is associated with lower bleeding events [60]. Among P2Y12 inhibitors, clopidogrel is preferred over prasugrel and ticagrelor because of the significantly higher bleeding risk associated with the latter agents [61]. Regarding the duration of triple antithrombotic therapy, current guidelines recommend a short course, typically up to 1 week, particularly in patients at HBR or when bleeding concerns outweigh ischemic risk, followed by dual therapy consisting of clopidogrel plus oral anticoagulation for up to 12 months, and lifelong oral anticoagulation thereafter [62]. Conversely, in selected patients with a particularly high bleeding risk, early discontinuation or omission of aspirin, with continuation of clopidogrel and oral anticoagulation alone, has been shown to provide a favorable safety profile without compromising ischemic outcomes, as demonstrated in a meta-analysis of three RCTs [60].

A specific challenge encountered during PCI is the inability to administer oral antiplatelet therapy, particularly in the setting of cardiogenic shock or when extracorporeal membrane oxygenation (ECMO) is required. In these situations, intravenous cangrelor, a reversible P2Y12 antagonist, represents a valuable alternative. Retrospective data in cardiogenic shock patients have not demonstrated any increase in bleeding compared to standard DAPT, while suggesting a reduction in MACE [63,64]. Prospective validation is awaited from the ongoing DAPT-SHOCK–AMI trial, which is comparing cangrelor versus ticagrelor in patients with myocardial infarction complicated by cardiogenic shock [65]. When transitioning to oral therapy, ticagrelor is the preferred agent, as it does not interact with cangrelor and can be administered during the cangrelor infusion [66]. It should be noted that in specific high-risk subgroups, such as patients aged ≥75 years or those requiring ECMO support, the substantially elevated bleeding risk must be carefully weighed against the ischemic benefit when considering potent antiplatelet agents such as ticagrelor or prasugrel [67].

Bleeding complications are frequent among patients requiring VA-ECMO after myocardial infarction [68]. The use of ticagrelor in this population is associated with increased bleeding risk compared to clopidogrel, while ischemic risk appears similar between the two agents. In cases where oral absorption is compromised, cangrelor represents a viable alternative. Case series have demonstrated a reduction in major bleeding with reduced-dose cangrelor (0.5 μg/kg/min) compared to standard dosing (0.75 μg/kg/min), which may represent a valuable strategy as a bridge to interventions such as left ventricular assist device implantation or when bleeding concerns are prominent [69]. However, no randomized controlled trials have been conducted on this topic.

## 4. Transfusion Strategies and Anemia Management

The SCAI recommends performing a complete blood count within 30 days to screen for and manage anemia when present. This is particularly important, as retrospective data have shown that hemoglobin levels <13 g/dL are associated with an increased risk of major bleeding, hospitalization, and mortality in both men and women. A recent meta-analysis of 44 studies including 170,914 patients undergoing PCI reported a prevalence of anemia of 16%, which was associated with a significantly increased risk of bleeding (RR 1.97, 95% CI 1.03–3.77) compared with non-anemic patients [70].

Current guidelines do not provide a clear recommendation on transfusion thresholds in ACS, reflecting ongoing uncertainty [11]. The Association for the Advancement of Blood & Biotherapies (AABB) recently suggested considering a more liberal transfusion strategy in patients with acute myocardial infarction, with a threshold around 10 g/dL with a low recommendation, as restrictive strategies (7–8 g/dL) may be associated with worse outcomes [71]. A systematic review of randomized trials, including MINT trial and REALITY trial, has not demonstrate a mortality difference between strategies, although a modest trend in favor of a liberal approach has been observed [72,73,74,75]. This has been reflected in the 2025 AHA recommendations for the management of ACS, which include a class IIb recommendation for the treatment of chronic anemia to reduce cardiovascular events [12]. Overall, the optimal transfusion threshold in ACS remains uncertain, and recommendations should be individualized.

A restrictive transfusion strategy (hemoglobin < 7 g/dL) is generally recommended in patients with chronic coronary syndrome. However, some authors suggest a higher threshold of 8 g/dL for individuals with pre-existing cardiovascular disease [76,77]. The relationship between iron deficiency, intravenous iron (IV) therapy, and ACS remains unclear, with limited evidence available. The INFERRCT trial (NCT05759078) will provide important insights into the role of IV iron (ferric carboxymaltose) in patients with recent myocardial infarction and iron deficiency.

Regarding erythropoietin use, it is not recommended in the cardiology setting. Indeed, the REVEAL trial randomized patients presenting with STEMI to receive either epoetin alfa or placebo following PCI. This strategy proved to be deleterious, with an increased risk of major adverse cardiovascular events, including death, stroke, myocardial infarction, and stent thrombosis, as well as a higher incidence of stent thrombosis [78]. Most data regarding the use of erythropoietin in combination with intravenous iron have been generated in surgical patients; however, to our knowledge, no such data are available in the setting of percutaneous interventions.

Patients undergoing PCI may also present with thrombocytopenia of varying severity and etiology. With the increasing prevalence of cancer and the widespread use of therapies associated with treatment-related thrombocytopenia, specific guidance has been developed to optimize antithrombotic management in this population. According to the SCAI recommendations, aspirin may be administered in patients with a platelet count ≥10 G/L, whereas dual antiplatelet therapy with clopidogrel is considered appropriate when the platelet count is ≥30 G/L and with prasugrel or ticagrelor when it is ≥50 G/L. PCI can generally be performed with a platelet count ≥30 G/L using a reduced dose of unfractionated heparin (30–50 U/kg), with regular monitoring of the activated clotting time (ACT) in patients with platelet counts ≤50 G/L. Prophylactic platelet transfusion is not recommended unless specifically advised by a hematologist [79].

These varying transfusion thresholds highlight the heterogeneity of the studied populations and support an individualized approach to transfusion decisions, balancing the potential benefits and risks for each patient.

## 5. Conclusions

Bleeding prevention has become a cornerstone of contemporary PCI management, particularly in patients at high bleeding risk. Current evidence supports a personalized approach that integrates bleeding risk stratification, radial access, optimized anticoagulation strategies, newer-generation drug-eluting stents, and abbreviated DAPT regimens to minimize bleeding complications while preserving ischemic protection.

Despite these advances, several important questions remain unanswered. Key areas of uncertainty include the optimal transfusion thresholds, the management of antithrombotic therapy in complex clinical scenarios, and the ideal balance between ischemic and bleeding risk in specific high-risk populations. Ongoing randomized clinical trials, including studies evaluating ultra-short DAPT after PCI in older patients (NCT07164859), abbreviated antiplatelet strategies following complex PCI in patients at HBR (BETA-DAPT; NCT07391358), and the role of drug-coated balloons in HBR patients (DCB-HBR; NCT05221931), are expected to provide important evidence to address these knowledge gaps.

Future research focusing on precision medicine, improved bleeding risk stratification, innovative coronary devices, and individualized antithrombotic strategies will further refine bleeding prevention and improve clinical outcomes for patients undergoing PCI.

## Figures and Tables

**Table 1:** DAPT strategy management.

Score	Purpose	Details
**BARC**	Standardized classification of bleeding events	Type 0 = no bleeding Type 1 = minor, no medical attention required Type 2 = requires medical evaluation but no severity criteria met Type 3 = major: 3A: any transfusion, hemoglobin drop of 3–5 g/dL 3B: hemoglobin drop of >5 g/dL, requirement of surgery or vasopressor 3C: intracranial or ocular hemorrhage Type 4 = CABG-related perioperative bleeding complications Type 5 = fatal bleeding (probable or definite)
**PRECISE-DAPT**	Estimate out-of-hospital bleeding risk in patients on DAPT prior to the procedure	Score ≥ 25 → high bleeding risk → shortened DAPT recommended Score < 25 → standard or prolonged DAPT (>12 months) without significant excess risk Should be calculated before every procedure Focused solely on bleeding risk (not ischemic risk)
**DAPT Score**	Guide the decision for prolonged DAPT by balancing ischemic and bleeding risk	Assessed after an initial DAPT period without major bleeding event Score ≥ 2 → prolonged DAPT is beneficial

## Data Availability

No new data were created or analyzed in this study. Data sharing is not applicable to this article.

## Abbreviations

The following abbreviations are used in this manuscript:

ACS	Acute coronary syndrome
CCS	Chronic coronary syndrome
DAPT	Dual antiplatelet therapy
HBR	High bleeding risk
PCI	Percutaneous coronary intervention
